# Association of *EGLN1* genetic polymorphisms with SpO_2_ responses to acute hypobaric hypoxia in a Japanese cohort

**DOI:** 10.1186/s40101-018-0169-7

**Published:** 2018-04-06

**Authors:** Yoshiki Yasukochi, Takayuki Nishimura, Midori Motoi, Shigeki Watanuki

**Affiliations:** 1 Department of Human Functional Genomics, Advanced Science Research Promotion Center, Organization for the Promotion of Regional Innovation, Mie University, 1577 Kurima-machiya, Tsu, Mie 514-8507 Japan; 2 Department of Public Health, Nagasaki University Graduate School of Biomedical Sciences, 1-12-4 Sakamoto, Nagasaki, 852-8523 Japan; 3 Department of Human Science, Kyushu University, 4-9-1 Shiobaru, Minami-ku, Fukuoka, 815-8540 Japan

**Keywords:** *EGLN1*, High-altitude adaptation, Hypobaric hypoxia, SNP, SpO_2_

## Abstract

**Background:**

Recent studies have explored various genetic and physiological factors related to high-altitude adaptation in highlander populations. However, the effects of single nucleotide polymorphisms (SNPs), influencing such adaptation, on physiological responses to hypobaric hypoxia have not been examined in lowlanders with lowlander ancestry. Thus, we aimed to investigate the association between SNPs around the *EGLN1* genomic region, possibly involved in high-altitude adaptation, and physiological changes to hypobaric hypoxia exposure in a cohort of Japanese lowlanders.

**Methods:**

Physiological data were obtained from 46 healthy Japanese male students under different atmospheric pressure conditions (equivalent to sea level and altitudes of 2500 and 4000 m). Genotypes of seven SNPs around *EGLN1* were determined in all subjects by PCR-direct sequencing or TaqMan SNP genotyping assay.

**Results:**

Results of the association study suggest that percutaneous arterial oxygen saturation (SpO_2_) responses of individuals with rs12097901 and rs2790859 alleles, whose frequencies are high in highlander populations (HL alleles), may be susceptible to acute hypobaric hypoxia. SpO_2_ levels of individuals with HL alleles were lower than those of individuals with non-HL alleles. At the same time, the subjects with HL alleles did not appear to have any remarkable hematological or pulmonary features that may counteract the low levels of SpO_2_. One may hypothesize that the low SpO_2_ levels in HL allele carriers could be a risk factor for acute mountain sickness in Japanese population.

**Conclusions:**

Our findings suggest that rs12097901 and rs2790859 genotypes affect SpO_2_ responses and may be associated with the susceptibility to acute hypobaric hypoxia in Japanese population.

**Electronic supplementary material:**

The online version of this article (10.1186/s40101-018-0169-7) contains supplementary material, which is available to authorized users.

## Background

Recent studies have identified single nucleotide polymorphisms (SNPs) related to physiological phenotypes that may be adaptive in high-altitude (hypobaric hypoxia) environments. In particular, physiological and genetic factors that contribute to high-altitude adaptations have been extensively studied in Tibetan, Ethiopian, and Andean highlander populations. Beall [1] suggested that physiological responses to hypobaric hypoxia stress were different in these three populations.

In recent years, genome-wide scans in Tibetan highlanders detected signals of recent positive selection in several chromosomal regions containing genes involved in the hypoxia-inducible factor (HIF) pathway, especially *EPAS1* and *EGLN1* [2–6]. HIFs play a central role in oxygen homeostasis and regulate erythropoiesis, angiogenesis, anaerobic metabolism, and glycolytic pathway [7, 8]. Under normoxic condition, prolyl hydroxylase domain-containing proteins [PHD1 (encoded by *EGLN2*), PHD2 (*EGLN1*), and PHD3 (*EGLN3*)], which are cellular oxygen sensors, hydroxylate specific proline residues of HIF-α (HIF-1α, HIF-2α, and HIF-3α), leading to the degradation of HIFs in the proteasome pathway involving the von Hippel-Lindau protein. Under hypoxia, HIF-α is not degraded and dimerizes with HIF-β, leading to the upregulation of proangiogenic molecules, such as the vascular endothelial growth factor and erythropoietin. Tibetan-specific *EPAS1* and *EGLN1* alleles were associated with low hemoglobin concentration in the population, and these alleles can be protective against chronic hypoxia caused by excessive erythrocytosis [2, 9].

A signal of recent positive selection in Andean and Tibetan populations has been commonly detected in a chromosomal region containing *EGLN1* that encodes the PHD2 protein [10]. This suggests that *EGLN1* may be a key factor for the high-altitude adaptation of modern humans. However, there have been no studies examining the effect of *EGLN1* genetic polymorphism on physiological responses of lowlanders with lowlander ancestry under hypoxia. Therefore, in the present study, we explored the relationship between *EGLN1* SNPs and physiological responses of Japanese lowlanders under acute hypobaric hypoxia.

## Methods

### Study subjects

A total of 46 healthy Japanese male students of Kyushu University (Fukuoka, Japan; mean age 22.5 ± 1.17 years) who had no clinical problems participated in the present study. None of them was a medium- or long-term highland resident within the last 2 months preceding the experiment. The subjects had been prohibited from taking exercise and drinking alcohol for 1 day, and from eating, smoking, and drinking caffeine for > 2 h before entering the hypobaric chamber. Their physical characteristics (mean values ± standard deviation) were as follows: height, 1.71 ± 0.05 m; weight, 61.1 ± 12.3 kg; body mass index (BMI), 20.8 ± 3.96 kg/m^2^; and body surface area, 1.66 ± 0.15 m^2^.

The study was approved by the Ethical Committee of Kyushu University Institutional Review Board for Human Genome/Gene Research and by the Committee on Human Research of Mie University Graduate School of Medicine.

### Physiological data collection

The experiment was carried out with the subjects wearing T-shirts and shorts. Measurement sensors were attached to the participants for approximately 30 min at 28 °C prior to the experiment, and then the subjects entered the hypobaric chamber. Physiological data, including oxygen consumption (VO_2_), carbon dioxide output (VCO_2_), minute ventilation (MV), percutaneous arterial oxygen saturation (SpO_2_), pulse rate (PR), and perfusion index (PI), were measured in the subjects that had rested in the sitting position. Hematological and pulmonary phenotypic data were independently collected in the following order of conditions: 765 Torr (equivalent to sea level) before decreasing the ambient atmospheric pressure, 562 Torr (equivalent to the altitude of 2500 m), 465 Torr (equivalent to 4000 m), and 765 Torr after pressure recovery. The detailed methods of the experiment and data collection are described elsewhere [11, 12].

### DNA extraction and SNP genotyping

Genomic DNA was extracted from subjects’ saliva using a Saliva DNA Isolation Kit (Norgen Biotek Corporation, Thorold, ON, Canada) according to the manufacturer’s protocol. Because the amount of genomic DNA was scarce, an Illustra GenomiPhi V2 DNA Amplification Kit (GE Healthcare Bio-Sciences Corp, NJ, USA) was used to perform whole-genome amplification. We selected seven SNPs (rs12097901, rs186996510, rs480902, rs479200, rs2808611, rs2790859, and rs2275279) around *EGLN1* to examine the association with time series variation of physiological measurements [at 0, 30 (equivalent to 2500 m), 60 (equivalent to 4000 m), and 90 min]. These SNPs have been previously suggested to be associated with high-altitude adaptation in highlander populations [3, 13, 14]. Of the seven selected SNPs, two (rs12097901 and rs186996510) are located in exon 1 of *EGLN1*. A DNA fragment covering a partial exon 1 sequence, including the two SNPs, was amplified by PCR using the following set of primers: EGLN1ex1-F, 5′-CAGTAACGGCCCCTATCTCTC-3′ (forward), and EGLN1ex1-R, 5′-TACTCGAGCGCCAGCTTC-3′ (reverse). PCR amplification was conducted in a 10-μL reaction mixture containing 0.2 μL (0.2 μM) each of forward and reverse primers, 5 μL of 2 × AmpliTaq Gold 360 Master Mix, 0.5 μL of the extracted DNA, and 4.1 μL of dH_2_O, using a TaKaRa PCR Thermal Cycler Dice (TaKaRa Bio). After incubation at 95 °C for 10 min, 30–35 cycles were performed as follows: 30 s at 95 °C, 30 s at 62 °C, 45 s at 72 °C, with a post-cycling extension at 72 °C for 7 min. Direct sequencing of the PCR products was performed by Eurofins Genomics (Tokyo, Japan). Five SNPs (rs480902, rs479200, rs2808611, rs2790859, and rs2275279) were genotyped by TaqMan SNP genotyping assay (Applied Biosystems, CA, USA).

### Statistical analyses

Genotype data of all subjects were converted into numeric data according to the dominant, additive, and recessive inheritance models. The dominant and recessive models were defined as “0, AA; 1, AB + BB” and “0, AA + AB; 1, BB” (A, major allele; B, minor allele), respectively, whereas the additive model was defined as “0, AA; 1, AB; 2, BB.” The significance of difference in physiological measurements between subjects with different genotypes in the dominant or recessive models was assessed using Welch’s *t*-test. To detect potential confounders, the generalized linear model (GLM) was applied to examine the correlation between anthropometric phenotypes and SpO_2_ in the additive model. A Gaussian distribution was selected for the family with an identity link in the model for the phenotypic data, because the SpO_2_ measurement data are continuous data. The GLM method showed no correlations between anthropometric phenotypes and SpO_2_ (*P* > 0.05), and thereby the GLM analysis without covariates was employed to test the relation among genotypes and the SpO_2_ measurements in the additive model. Two-way analysis of variance (ANOVA) was conducted using time (the experimental conditions) and genotype as independent variables and the repeated measurements as dependent variables. The statistical tests described above were performed using R software version 3.4.2 [15] via RStudio version 1.1.383 [16]. Linear regression analysis was conducted to examine the relationships between SpO_2_ and several hematological and pulmonary phenotypes. For regression analysis, we applied the analysis of covariance (ANCOVA) to test the differences in the regression coefficients (i.e., slope of regression line) between genotypes and in the adjusted mean values (i.e., the intercept of the regression line) [17].

### Population genetics

Haplotype phase and linkage disequilibrium (LD) between SNPs were estimated using IMPUTE2 version 2.3.2 [18] and Haploview version 4.2 [19] programs, respectively. We estimated D′ values among SNPs using R package “genetics” [20]. The LDs between pairs of the *EGLN1* SNPs in four ethnic groups (East Asian, South Asian, European, and African populations) from the 1000 Genomes Project (http://www.internationalgenome.org/, [21]) were surveyed, employing LDlink web-based tools (https://analysistools.nci.nih.gov/LDlink/, [22]). The categories of four ethnic groups are listed at http://www.internationalgenome.org/data-portal/population. A large integrated variant dataset of JPT (Japanese in Tokyo, Japan) from the 1000 Genomes Project was obtained through the 1000 Genomes Browser (http://phase3browser.1000genomes.org/). Allele frequency data were obtained through the Ensembl genome browser (http://www.ensembl.org, [23]). The allele whose frequency was high in African populations was defined as the ancestral allele. Significant deviations from the Hardy-Weinberg equilibrium (HWE) were tested by Fisher’s exact test, using PLINK 1.90 (http://zzz.bwh.harvard.edu/plink/, [24]). Network and neighbor-joining (NJ) tree [25] of the estimated haplotypes were constructed with the neighbor-net method [26] by using Splits-tree4 ver. 4.14.4 [27] and with p-distance by using MEGA version 7 [28], respectively.

## Results

### Genotypes of seven SNPs around *EGLN1* in 46 Japanese subjects

We genotyped the seven selected SNPs around *EGLN1* in 46 healthy Japanese males (Table 1). Frequencies of derived alleles (~ 0.6) at two SNPs (rs480902 and rs2808611) were higher than those of ancestral alleles (~ 0.4). In addition, frequency data of derived alleles in African populations from the Ensembl genome browser were approximately 0.4, suggesting that the frequencies of the derived alleles may have increased after Out-of-Africa. Derived alleles of three SNPs (rs479200, rs12097901, and rs186996510) or ancestral alleles of the remaining four SNPs were frequently observed in highlander populations [3, 13, 14]. We refer to the allele whose frequency is high in highlander populations as “HL allele” (Table 1).

**Table 1:** Summary of SNPs examined in the present study

RefSNP ID	Position^a^	Region	*N*	Allele frequency^b^		HL allele^d^	Allele frequency in highlanders^d^	H_obs_	H_exp_	HWE (*P* value)^e^
				Ancestral^c^	Derived^c^					
rs480902	1: 231,395,881	*EGLN1* intron 1	46	T: 0.413 (38)	C: 0.587 (54)	T	0.71	0.565	0.485	0.37
rs479200	1: 231,408,034	*EGLN1* intron 1	46	T: 0.609 (56)	C: 0.391 (36)	C	0.71	0.478	0.476	1.00
rs2808611	1: 231,412,734	*EGLN1* intron 1	46	G: 0.424 (39)	A: 0.576 (53)	G	NA	0.500	0.488	1.00
rs12097901	1: 231,421,509	*EGLN1* exon 1 (C127S)^f^	46	G: 0.533 (49)	C: 0.467 (43)	C	0.90	0.457	0.498	0.56
rs186996510	1: 231,421,877	*EGLN1* exon 1 (D4E)^f^	46	C: 0.967 (89)	G: 0.033 (3)	G	0.81	0.022	0.063	*0.03*
rs2790859	1: 231,465,611	Intergenic region	46	T: 0.620 (57)	C: 0.380 (35)	T	0.76	0.543	0.471	0.37
rs2275279	1: 231,591,348	*TSNAX-DISC1*	46	A: 0.663 (61)	T: 0.337 (31)	A	0.69	0.283	0.447	*0.02*

Abbreviations: *SNP* single nucleotide polymorphism, *H*_*obs*_ observed heterozygosity, *H*_*exp*_ expected heterozygosity, *HWE* Hardy-Weinberg equilibrium, *NA* not available

^a^Position in NCBI build GRCh38

^b^Values indicate allele frequencies, and the observed numbers are indicated in parentheses

^c^The allele whose frequency was high in African populations was defined as the ancestral allele, according to allele frequency data obtained through the Ensembl genome browser (http://www.ensembl.org, [23])

^d^Allele frequencies are high in highlander populations [3, 13, 14]

^e^Statistically significant *P* values (*P* < 0.05, by Fisher’s exact test) are shown in italics

^f^Amino acid substitution

**Fig. 1:**
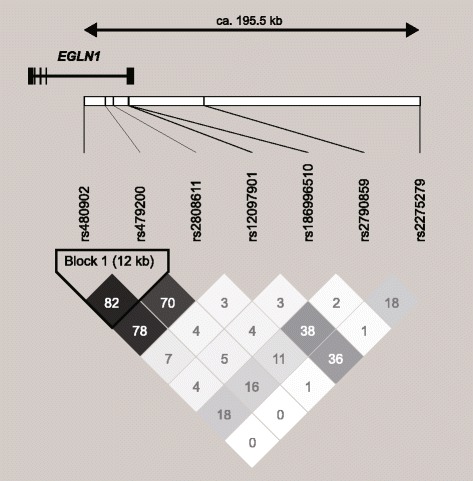
The physical position and linkage disequilibrium of seven SNPs across a ~ 195.5-kb genomic region at 1q42.2 in 46 Japanese subjects. The diagram was created by Haploview version 4.2. The number in a diamond represents *r*^2^ value (× 100). The haplotype block was defined by the method of Gabriel et al. [36]

The target seven SNPs are closely located in the chromosomal region 1q42.2. We thus examined LDs between pairs of these SNPs (Fig. 1). The LDs between three SNPs (rs480902, rs479200, and rs2808611) were relatively strong (D′ = 0.90–0.95, *r*^2^ = 0.70–0.82). In African populations, from the 1000 Genomes Project database, *r*^2^ values between pairs of the three SNPs were low (*r*^2^ = 0.16–0.34), although D′ values were relatively high (D′ = 0.43–0.99), according to LDmatrix, an LDlink application. In three ethnic groups (East Asians, South Asians, and Europeans), the *r*^2^ and D′ values were higher than those in the African populations (D′ = 0.94–0.95 and *r*^2^ = 0.82–0.98 for East Asians; D′ = 0.98–1.00 and *r*^2^ = 0.73–0.84 for South Asians; D′ = 0.96–1.00 and *r*^2^ = 0.51–0.87 for Europeans). These results suggest that the strong LDs may have been established since the Out-of-Africa event. We additionally examined LDs between pairs of 1504 biallelic sites across a ~ 399-kb genomic region at 1q42.2, based on datasets from JPT in the 1000 Genomes Project database. The LD plot indicated that the three SNPs (rs480902, rs479200, and rs2808611) were in a large LD block while the other SNPs examined were located outside the LD block in the chromosomal region (Additional file 1: Figure S1). The values of *r*^2^ between pairs of the remaining four SNPs examined in the present study were less than 0.4 (*r*^2^ = 0.00–0.38), whereas the D′ values were relatively high (D′ = 0.50–1.00).

Among the seven SNPs examined, two (rs2275279 and rs186996510) showed significant deviations from HWE (*P* = 0.02–0.03) (Table 1). Thus, we removed these SNPs from further analyses. The mean observed and expected heterozygosities for the remaining five SNPs among the 46 subjects were 0.509 and 0.484, respectively. Based on Bonferroni’s correction, *P* values of < 0.01 (0.05/5 SNPs examined) were considered statistically significant for further analyses.

### Association of physiological responses to acute hypobaric hypoxia with the genotypes of five SNPs around *EGLN1*

**Fig. 2:**
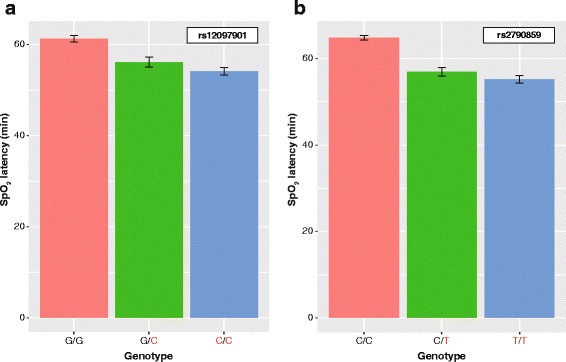
SpO_2_ latency at 28 °C in individuals with different genotypes of rs12097901 (**a**) and rs2790859 (**b**). The ordinate axis represents SpO_2_ latency (min) in each genotype. Red characters represent highlander alleles. Data are presented as the mean ± standard error of the mean

We examined the relationships between genotypes of the five SNPs around *EGLN1* and physiological responses to acute hypobaric hypoxia exposure (at 0, 30, 60, and 90 min) in the three inheritance models. Time series variations of pulmonary and hematological traits (VO_2_, VCO_2_, MV, SpO_2_, PR, and PI) and SpO_2_ latency (latency in the first reading and time to stabilization of SpO_2_) in individuals with different genotypes of each SNP are shown in Table 2 and Additional file 2: Table S1. Through the GLM analysis, the rs12097901 genotype showed a significant association with SpO_2_ latency (*P* = 0.006) in the additive model, whereas the rs2790859 genotype was related to the latency with borderline significance (*P* = 0.014) (Table 2 and Fig. 2). In addition, the significant relations of SpO_2_ latency to rs12097901 and rs2790859 genotypes were shown in the dominant and recessive models, respectively (*P* = 0.003). SpO_2_ latencies of individuals with HL alleles of these SNPs were shorter than those of individuals with non-HL alleles. The differences of SpO_2_ latencies may be attributable to effects on physiological reaction time to acute hypobaric hypoxia exposure. In the present study, three of the 46 subjects examined showed high BMI values (> 30 kg/m^2^). It has been reported that BMI may influence SpO_2_ levels [29]. However, the corresponding SpO_2_ values were not extremely high (Additional file 3: Figure S2). Thus, these subjects were included for further analyses.

**Table 2:** Time series variations of SpO_2_ in individuals with different genotypes

RefSNP ID	Position^a^	Genotype^b^	2*N*^c^	SpO_2_^d^				SpO_2_ latency^e^	*P* value^f^
				0 min	30 min	60 min	90 min		
rs480902	1: 231,395,881	CC	14	98.3 ± 0.24	93.6 ± 0.35	86.0 ± 0.84	97.0 ± 0.30	58.1 ± 1.61	0.367
		*T*C	26	98.5 ± 0.19	94.1 ± 0.31	86.2 ± 0.57	97.6 ± 0.29	57.2 ± 1.46	
		*TT*	6	98.2 ± 0.67	93.7 ± 0.41	85.7 ± 0.56	97.2 ± 0.63	55.0 ± 2.27	
rs479200	1: 231,408,034	TT	17	98.3 ± 0.22	93.6 ± 0.30	86.2 ± 0.73	97.1 ± 0.27	57.5 ± 1.52	0.527
		T*C*	22	98.5 ± 0.22	94.2 ± 0.36	86.1 ± 0.66	97.5 ± 0.34	57.5 ± 1.63	
		*CC*	7	98.3 ± 0.57	93.6 ± 0.38	85.8 ± 0.48	97.4 ± 0.55	55.1 ± 1.92	
rs2808611	1: 231,412,734	AA	15	98.3 ± 0.25	93.7 ± 0.32	86.1 ± 0.80	97.0 ± 0.30	58.1 ± 1.52	0.766
		*G*A	23	98.5 ± 0.21	94.0 ± 0.36	86.0 ± 0.63	97.5 ± 0.31	57.4 ± 1.55	
		*GG*	8	98.4 ± 0.50	93.9 ± 0.34	86.2 ± 0.58	97.5 ± 0.57	54.8 ± 2.25	
rs12097901	1: 231,421,509	GG	14	99.0 ± 0.10	94.5 ± 0.31	87.2 ± 0.60	97.2 ± 0.33	61.2 ± 1.35	0.006
		*C*G	21	98.2 ± 0.20	93.4 ± 0.32	85.5 ± 0.68	97.3 ± 0.30	56.1 ± 1.56	
		*CC*	11	98.1 ± 0.46	93.9 ± 0.47	85.8 ± 0.79	97.6 ± 0.51	54.1 ± 1.76	
rs2790859	1: 231,465,611	*TT*	16	98.0 ± 0.33	93.5 ± 0.35	85.4 ± 0.71	97.3 ± 0.35	55.2 ± 1.44	0.014
		*T*C	25	98.5 ± 0.17	94.0 ± 0.30	86.1 ± 0.56	97.5 ± 0.28	56.9 ± 1.38	
		CC	5	99.1 ± 0.19	94.6 ± 0.55	88.2 ± 0.77	96.6 ± 0.61	64.8 ± 1.71	

Abbreviation: *SpO*_*2*_ percutaneous arterial oxygen saturation

^a^Position in NCBI build GRCh38

^b^Highlander alleles are shown in italics (see Table 1)

^c^The number of chromosomes

^d^Quantitative data are presented as the mean ± standard error of the mean

^e^Latency in the first reading and time to stabilization of SpO_2_

^f^*P* values estimated by the GLM analysis for SpO_2_ latency

**Fig. 3:**
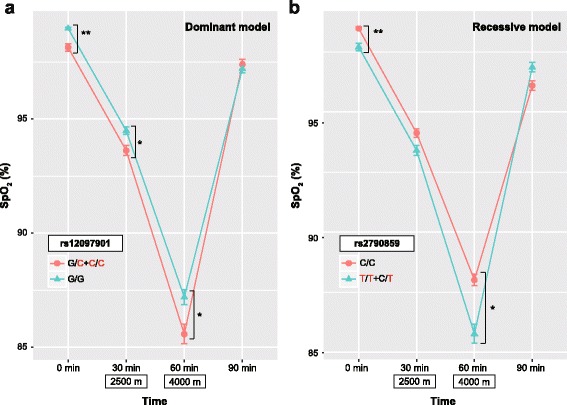
Time series variations of SpO_2_ at 28 °C in two groups of subjects differentiated by rs12097901 (**a**) and rs2790859 (**b**) genotypes. The ordinate axis represents SpO_2_ and the abscissa axis represents time (min): 30 and 60 min are equivalent to altitudes of 2500 and 4000 m, respectively. Red characters represent highlander alleles. Line colors represent each group of genotypes. Data are presented as the mean ± standard error of the mean. ^*^P < 0.05; ^**^P < 0.01 (by Welch’s *t*-test)

In the dominant model, the mean SpO_2_ values from 0 to 60 min were affected by rs12097901 genotype: the mean values in the combined groups of homozygotes with derived allele (HL allele) and heterozygotes with ancestral and derived alleles were lower than those in homozygotes with ancestral allele (Table 2 and Fig. 3a). The difference in SpO_2_ values at 0 min between the groups was significant (*P* = 0.003, by Welch’s *t*-test). In contrast, the mean SpO_2_ value at 90 min in the combined group was slightly higher than that in homozygotes with ancestral allele. Although the interaction between time and genotype was not statistically significant, the *P* value was ~ 0.05 (*F*_(3, 132)_ = 2.6, *P* = 0.052, by two-way ANOVA).

In the recessive model, rs2790859 showed a similar effect to that of rs12097901 (Table 2 and Fig. 3b). The mean SpO_2_ values from 0 to 60 min in the combined group of homozygotes with ancestral allele (HL allele) and heterozygotes with ancestral and derived alleles were lower in homozygotes with derived allele. The difference in SpO_2_ values at 0 min was statistically significant (*P* = 0.008), and the interaction between time and genotype was borderline significant (*F*_(3, 132)_ = 3.6, *P* = 0.015): the mean SpO_2_ value at 90 min in individuals with HL alleles was higher than that in individuals with non-HL allele.

### Effects of estimated haplotypes or diplotypes on SpO_2_ values

**Fig. 4:**
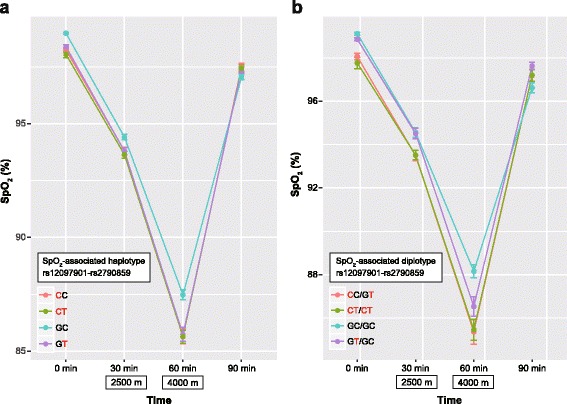
Time series variations of SpO_2_ at 28 °C in groups of haplotypes (**a**) and diplotypes (**b**) estimated by using rs12097901 and rs2790859 genotype frequencies. Estimated diplotypes with a sample size of < 5 were removed. The ordinate axis represents SpO_2_ and the abscissa axis represents time (min): 30 and 60 min are equivalent to altitudes of 2500 and 4000 m, respectively. Red characters represent highlander alleles. Line colors represent each group of haplotypes or diplotypes. Data are presented as the mean ± standard error of the mean

Using IMPUTE2 program, we estimated phased haplotypes comprising the five SNPs examined in all subjects. Haplotype and diplotype frequencies are shown in Additional file 4: Table S2. Here, we focus on haplotypes of rs12097901 and rs2790859 related to SpO_2_ (Table 3). Haplotype “CT” (from left, rs12097901 and rs2790859) comprised HL alleles (HL haplotype), whereas haplotype “GC” included all non-HL alleles (non-HL haplotype). In the 46 subjects, the proportion of haplotype “GT” was the highest (32%), followed by “CT” (30%), “GC” (22%), and “CC” (16%). The proportions of diplotypes “CT/CT” (HL diplotype) and “GC/GC” (non-HL diplotype) were 20 and 11%, respectively. We compared time series variations of SpO_2_ levels among individuals with different haplotypes (Fig. 4a) and diplotypes (Fig. 4b). The mean SpO_2_ values of non-HL haplotype “GC” from 0 to 60 min were higher than those of other haplotypes, whereas SpO_2_ levels of non-HL haplotype at 90 min were lower than those in individuals with other haplotypes. Similarly, SpO_2_ values of diplotypes in subjects with non-HL haplotypes (“GC/GC” and “GT/GC”) from 0 to 60 min were higher than those in subjects with other diplotypes without non-HL haplotypes. These results were consistent with the results of the genotype-based association studies.

**Table 3:** Haplotype and diplotype frequencies of rs12097901 and rs2790859 in 46 Japanese subjects

Haplotype^a^	Observed number	Frequency	Diplotype^a^	Observed number	Frequency
G*T*	29	0.32	*C*C/G*T*	13	0.28
*CT*	28	0.30	*CT*/*CT*	9	0.20
GC	20	0.22	G*T*/GC	6	0.13
*C*C	15	0.16	GC/GC	5	0.11
			*CT*/GC	4	0.09
			*CT*/G*T*	4	0.09
			G*T*/G*T*	3	0.07
			*C*C/*CT*	2	0.04

Haplotype consists of two candidate SNPs related to SpO_2_ (from left, rs12097901 and rs2790859)

^a^Highlander alleles are shown in italics (see Table 1)

**Fig. 5:**
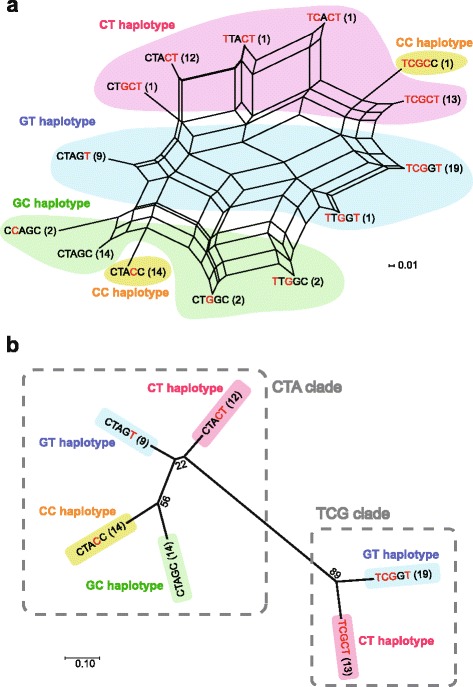
Neighbor-net network (**a**) and neighbor-joining tree (**b**) of the estimated haplotypes of five *EGLN1* SNPs. The observed number of haplotypes is indicated in parentheses. The neighbor-joining tree was constructed after removing haplotypes with a sample size of < 5, and the number of internal nodes represents bootstrap values (1000 replications). Red characters represent highlander alleles of each SNP. CT, GT, GC, and CC haplotypes were defined by rs12097901 and rs2790859 alleles. CTA and TCG clades were defined by rs480902, rs479200, and rs2808611 alleles

Using estimated haplotype sequences comprising all SNPs examined, we generated a haplotype network (Fig. 5a). This network indicated that HL or non-HL haplotypes of two SpO_2_-associated SNPs (i.e., rs12097901 and rs2790859) and the other three SNPs were mixed with each other, suggesting that the chromosomal region around *EGLN1* has undergone recombination events. To simplify the relationships between the haplotypes, an unrooted NJ tree was constructed after removing haplotypes with a sample size of < 5 (Fig. 5b). The topology of this tree showed that all haplotype groups of SpO_2_-associated SNPs (GT, CT, GC, and CC) were included in CTA clade with non-HL alleles of the other three SNPs (from left, rs480902, rs479200, and rs2808611), whereas TCG clade with HL alleles had two SpO_2_-associated haplotypes only (CT and GT). Thus, the diversity of CTA lineage appears to be higher than that of TCG lineage.

### Relationships between SpO_2_ and other hematological or pulmonary phenotypes

**Fig. 6:**
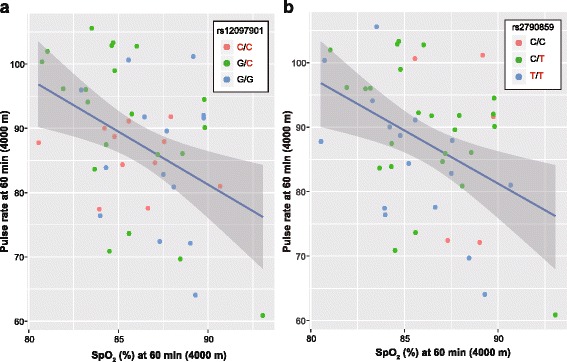
Relationship between SpO_2_ and pulse rate at 60 min (equivalent to the altitude of 4000 m) for rs12097901 (**a**) and rs2790859 (**b**). Red characters represent highlander alleles. Blue line and gray band represent a regression line and its 95% confidence interval, respectively. Circle colors indicate genotypes of each SNP. The mean slope of the regression line was − 1.65. Linear regression analysis showed significant correlation (*r*^2^ = 0.16, *P* = 0.003). ANCOVA showed no significant differences in the regression coefficient between genotypes (*F*_(2, 40)_ = 1.31, *P* = 0.282 for rs12097901; *F*_(2, 40)_ = 0.19, *P* = 0.824 for rs2790859) and in the adjusted mean values (*F*_(2, 42)_ = 0.61, *P* = 0.546 for rs12097901; *F*_(2, 42)_ = 2.17, *P* = 0.127 for rs2790859)

We applied linear regression model to examine correlations between SpO_2_ and the following hematological or pulmonary traits: MV, PR, PI, and respiratory exchange ratio (*R* = VCO_2_/VO_2_). This analysis indicated that SpO_2_ levels negatively correlated with PR (*r*^2^ = 0.16, *P* = 0.003; Fig. 6), whereas there was no significant correlation between SpO_2_ and other phenotypes examined (Additional file 5: Figure S3, Additional file 6: Figure S4 and Additional file 7: Figure S5). For regression analysis, we performed ANCOVA to assess the differences in the regression coefficients between rs12097901 or rs2790859 genotypes and in the adjusted mean values. However, there were no significant differences in either slope (*F*_(2, 40)_ = 0.19–2.20, *P* = 0.124–0.825 for rs12097901; *F*_(2, 40)_ = 0.04–0.98, *P* = 0.384–0.961 for rs2790859) or intercept (*F*_(2, 42)_ = 0.59–2.97, *P* = 0.062–0.557 for rs12097901; *F*_(2, 42)_ = 0.11–2.17, *P* = 0.127–0.894 for rs2790859) of the regression lines (Fig. 6 and Additional file 5: Figure S3, Additional file 6: Figure S4 and Additional file 7: Figure S5).

## Discussion

In the present study, the HL alleles of rs12097901 and rs2790859 were associated with a shorter SpO_2_ latency, compared to that in subjects harboring non-HL alleles. In addition, the recovery of SpO_2_ from 60 to 90 min of the hypobaric hypoxia experiment was greater in HL allele carriers than in subjects with non-HL alleles. These results suggest that HL allele carriers are more susceptible to acute hypobaric hypoxia. SpO_2_ levels in individuals with HL alleles from 0 to 60 min were lower than those in subjects with non-HL alleles. It is possible that the effect of HL alleles on SpO_2_ levels under hypobaric hypoxia conditions may lead to the development of acute mountain sickness (AMS). AMS is defined by the presence of headache and at least one of the following symptoms: gastrointestinal upset (anorexia, nausea, or vomiting), fatigue or weakness, dizziness or lightheadedness, and difficulty falling asleep [30]. Although there is no reliable diagnostic modality or physical finding, arterial oxygen saturation (SaO_2_) could be a simple indicator of AMS development [31]. In the present study, the *r*^2^ value between rs12097901 and rs2790859 was low (*r*^2^ = 0.38), whereas the D′ value was high (D′ = 0.85). D′ tends to easily show high values in the absence of any of the four possible haplotypes, whereas *r*^2^ tends to show low values when SNPs with rare alleles are included in haplotypes being examined [32]. In addition, a small sample size could lead to biased LD estimates. Therefore, the effective independence of each SNP from SpO_2_ variation remains unclear. Irrespective of the independence, genotyping of the two SNPs related to SpO_2_ might be useful for the prevention and early diagnosis of AMS in Japanese population.

A previous study on comparisons of SaO_2_ levels among three highlander populations (Tibetan, Ethiopian, and Andean populations) showed the lowest SaO_2_ levels in Tibetans [1]. However, even though SaO_2_ levels of Tibetans are low, they maintain high resting ventilation [33] and may counteract low levels of arterial oxygen by high levels of circulating nitric oxide that increases blood flow [9]. In the present study, however, we did not reveal any remarkable hematological or pulmonary features in Japanese individuals with HL alleles of the two candidate SNPs related to SpO_2_. One may hypothesize that HL alleles in Japanese population are not adaptive to acute and short hypobaric hypoxia. At the same time, HL allele might be a risk factor for acute hypobaric hypoxia in Japanese population, because Japanese individuals with the HL allele do not appear to have remarkable hematological or pulmonary features that can possibly counteract low levels of SpO_2_. To validate this hypothesis, further analyses are required to examine effects of SNPs on long-term acclimatization and hemoglobin concentrations in lowlanders with lowlander ancestry under hypoxia.

In Indian lowlander populations, rs480902 and rs479200 were associated with the expression level of *EGLN1* and SaO_2_ levels [34]. In addition, rs480902 also showed a significant relation to SaO_2_ in Han Chinese AMS patients [35]. However, such association for rs480902 and rs479200 was not observed in the present study. The discrepancy of the relations between SNPs and the phenotypes might be due to differences in the genetic or physiological background among ethnic groups. Alternatively, given that the association of rs480902 with SaO_2_ was not observed in the Han AMS patients, but not in the control group, the significance of the association might be detectable in Japanese AMS patients. The T allele could be a risk factor for the AMS, according to the results of Buroker et al. [35]. The frequencies of this allele tend to be high in highlanders and are consistent with low SpO_2_ levels of HL alleles. Further investigation is required to determine the association between rs480902 and SpO_2_ in Japanese populations.

The haplotype network analysis suggested that recombination event had occurred around the *EGLN1* genomic region. The haplotype phase of the three SNPs with strong LDs (rs480902, rs479200, and rs2808611) was relatively conserved, and in the network, all SpO_2_-associated haplotype lineages were included in only the CTA lineage of the three SNPs. Furthermore, *r*^2^ values between rs2808611 and rs12097901 were extremely low (*r*^2^ = 0.03) for the physical distance. These results suggest that recombination breakpoints may be located around the boundary between these two SNPs. In fact, according to datasets of JPT in the 1000 Genomes Project database, the three SNPs with strong LDs were in an LD block outside rs12097901. In the haplotype network, the CTA clade included CTA, CTG, and CCA haplotypes, whereas TCG haplotype was only in the TCG clade. The difference in the number of nucleotides between CTA and the other haplotypes in the clade was only one, and the mutations (CTA → CTG and CTA → CCA) were of the transition type (In general, the frequency of transition mutation is higher than that of transversion mutation). Furthermore, the observed number of CTG and CCA haplotypes was small (≤ 2). Consequently, CTG and CCA haplotypes may have arisen from the CTA haplotype by a single nucleotide mutation and have been maintained in the gene pool of the Japanese cohort. The paraphyletic clades of CTA haplotypes might be formed after recombination has occurred in the region including the other two SNPs (rs12097901 and rs2790859). Although we have not revealed the association between haplotypes consisting of the three SNPs with strong LDs and pulmonary or hematological phenotypes, the divergence of CTA and TCG haplotypes might influence the differences in the function of phenotypes that depend on the HIF pathway.

In Tibetan populations, rs12097901 and rs186996510 are in a strong LD because of strong positive selection (rapid expansion of the HL haplotype), and these SNPs may have epistatic effects on enzymatic activity [14]. In the Japanese cohort examined, the two SNPs were not in LD (*r*^2^ = 0.03), and there were two individuals with the HL haplotype (heterozygote and homozygote of the HL haplotype). However, these individuals did not show remarkably high or low quantitative values of pulmonary and hematological phenotypes, although the homozygote showed low SpO_2_ values. Given that the nucleotide substitution at rs12097901 alters an amino acid of the PHD2 protein (encoded by *EGLN1*), and that pulmonary and hematological phenotypes are determined by polygenic inheritance, rs12097901 genotypes possibly have some effects on the enzymatic activity even if the effects are small.

There were several limitations in the present study. First, the effects of long-term acclimatization of the lowlander population on SpO_2_ changes remain unclear. Second, the sample size is not sufficient to detect relations between SNPs and phenotypes with a high statistical power, although it is difficult to obtain data from a large number of subjects due to experimental limitations. Third, the functional relevance of the two candidate SNPs to SpO_2_ variations remains unclear. A number of SNPs in various genes can contribute to high-altitude adaptation in highlanders. In future, genome-wide association study and functional analysis are required to evaluate the overall effect of genetic polymorphisms on inter-individual differences in physiological responses to hypobaric hypoxia.

## Conclusions

We showed that rs12097901 and rs2790859 were associated with SpO_2_ variations and SpO_2_ latency in an acute hypobaric hypoxia experiment in a Japanese cohort. SpO_2_ values of individuals with HL alleles of these two SNPs were lower than those of individuals with non-HL alleles. It is possible that HL alleles may confer susceptibility to acute hypobaric hypoxia in Japanese populations.

## Additional files

Additional file 1: **Figure S1.** Linkage disequilibrium plot of 1504 biallelic sites across a ~ 399-kb genomic region at 1q42.2, based on datasets from JPT (Japanese in Tokyo, Japan) in the 1000 Genomes Project database. The position of SNPs examined is shown by the green arrow. The haplotype block was defined by the method of Gabriel et al. [36]. The genetic variants with the minor allele frequency of < 0.01 were removed from the analysis. (PDF 219 kb)

Additional file 2: **Table S1.** Time series variations of pulmonary and hematological phenotypes in individuals with different genotypes. (XLSX 15 kb)

Additional file 3: **Figure S2.** Relationship between BMI and SpO_2_ at sea level in 46 Japanese male students. (PDF 101 kb)

Additional file 4: **Table S2.** Haplotype and diplotype frequencies of five SNPs around *EGLN1* in 46 Japanese subjects. (PDF 71 kb)

Additional file 5: **Figure S3.** Relationship between SpO_2_ and minute ventilation at 60 min (equivalent to 4000 m) for rs12097901 (a) and rs2790859 (b). Red characters represent highlander alleles. Blue line and gray band represent a regression line and its 95% confidence interval, respectively. Circle colors indicate genotypes of each SNP. The mean slope of the regression line was − 0.018. Linear regression analysis showed no significant correlation (*r*^2^ = 0.001, *P* = 0.818). ANCOVA also showed no significant differences in the regression coefficient between genotypes (*F*_(2, 40)_ = 1.21, *P* = 0.309 for rs12097901; *F*_(2, 40)_ = 0.04, *P* = 0.961 for rs2790859) and in the adjusted mean values (*F*_(2, 42)_ = 2.97, *P* = 0.062 for rs12097901; *F*_(2, 42)_ = 0.11, *P* = 0.894 for rs2790859). (PDF 230 kb)

Additional file 6: **Figure S4.** Relationship between SpO_2_ and perfusion index at 60 min (equivalent to 4000 m) for rs12097901 (a) and rs2790859 (b). Red characters represent highlander alleles. Blue line and gray band represent a regression line and its 95% confidence interval, respectively. Circle colors indicate genotypes of each SNP. The mean slope of the regression line was 0.011. Linear regression analysis showed no significant correlation (*r*^2^ = 0.011, *P* = 0.481). ANCOVA also showed no significant differences in the regression coefficient between genotypes (*F*_(2, 40)_ = 2.20, *P* = 0.124 for rs12097901; *F*_(2, 40)_ = 0.69, *P* = 0.509 for rs2790859) and in the adjusted mean values (*F*_(2, 42)_ = 1.29, *P* = 0.285 for rs12097901; *F*_(2, 42)_ = 1.08, *P* = 0.348 for rs2790859). (PDF 227 kb)

Additional file 7: **Figure S5.** Relationship between SpO_2_ and respiratory exchange ratio at 60 min (equivalent to 4000 m) for rs12097901 (a) and rs2790859 (b). Red characters represent highlander alleles. Blue line and gray band represent a regression line and its 95% confidence interval, respectively. Circle colors indicate genotypes of each SNP. The mean slope of the regression line was 0.002. Linear regression analysis showed no significant correlation (*r*^2^ = 0.012, *P* = 0.474). ANCOVA also showed no significant differences in the regression coefficient between genotypes (*F*_(2, 40)_ = 0.19, *P* = 0.825 for rs12097901; *F*_(2, 40)_ = 0.98, *P* = 0.384 for rs2790859) and in the adjusted mean values (*F*_(2, 42)_ = 0.59, *P* = 0.557 for rs12097901; *F*_(2, 42)_ = 0.17, *P* = 0.840 for rs2790859). (PDF 215 kb)

## Abbreviations

AMS: Acute mountain sickness
ANCOVA: Analysis of covariance
ANOVA: Analysis of variance
BMI: Body mass index
GLM: Generalized linear model
HIF: Hypoxia-inducible factor
HL allele: Highlander allele
HWE: Hardy-Weinberg equilibrium
JPT: Japanese in Tokyo, Japan
LD: Linkage disequilibrium
MV: Minute ventilation
NJ: Neighbor-joining
PHD: Prolyl hydroxylase domain-containing
PI: Perfusion index
PR: Pulse rate
SaO_2_: Arterial oxygen saturation
SNP: Single nucleotide polymorphism
SpO_2_: Percutaneous arterial oxygen saturation
VCO_2_: Carbon dioxide output
VO_2_: Oxygen consumption
