# Supplementary material for: Association of EGLN1 genetic polymorphisms with SpO2 responses to acute hypobaric hypoxia in a Japanese cohort
Source: J Physiol Anthropol. 2018 Apr 6;37:9. doi: 10.1186/s40101-018-0169-7 (PMC5889538; doi:10.1186/s40101-018-0169-7)
Supplement: Supplementary file 1 — Figure S1. Linkage disequilibrium plot of 1504 biallelic sites across a ~ 399-kb genomic region at 1q42.2, based on datasets from JPT (Japanese in Tokyo, Japan) in the 1000 Genomes Project database. The position of SNPs examined is shown by the green arrow. The haplotype block was defined by the method of Gabriel et al. [36]. The genetic variants with the minor allele frequency of < 0.01 were removed from the analysis. (PDF 219 kb) [file 40101_2018_169_MOESM1_ESM.pdf]

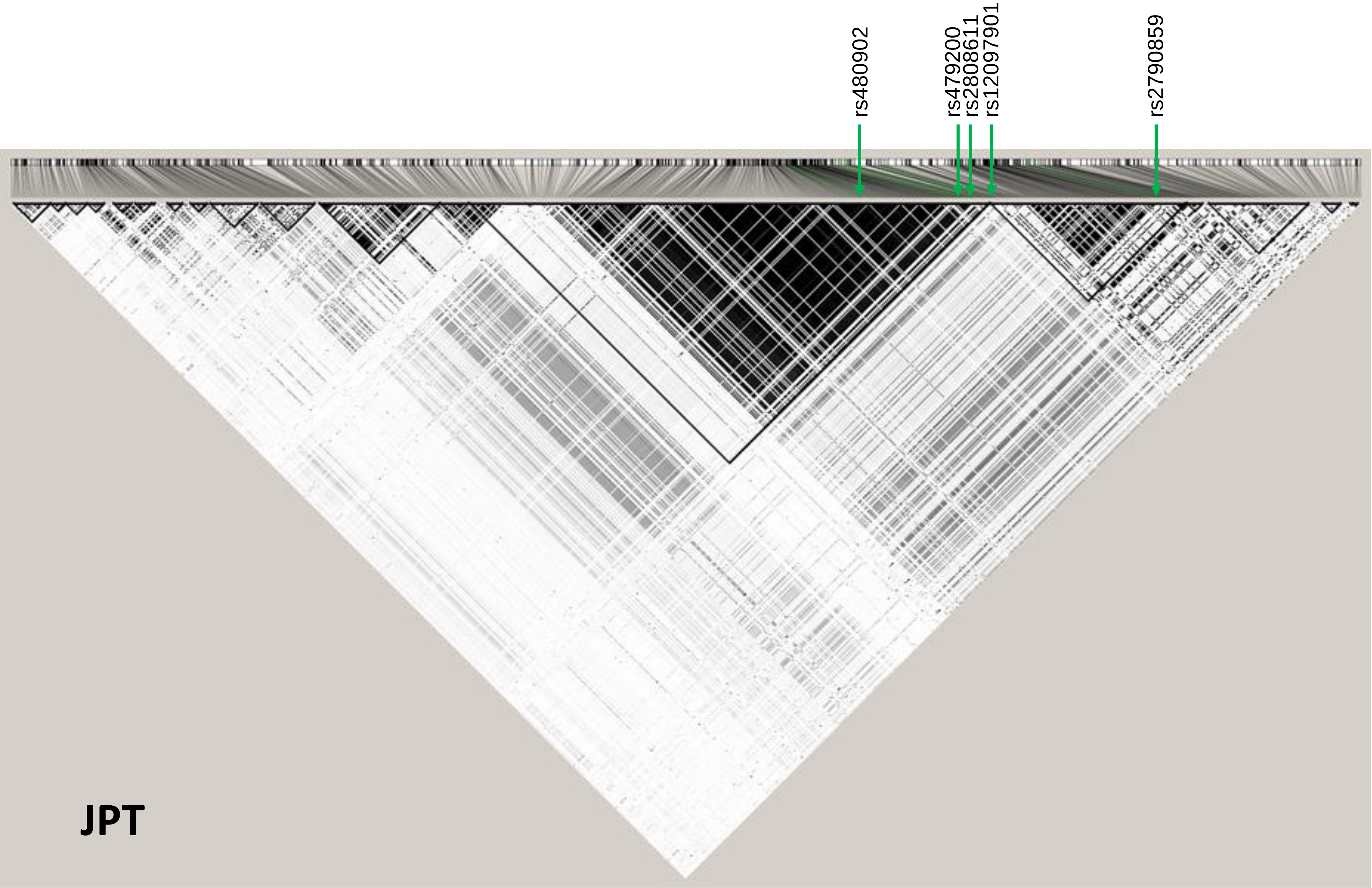

Fig. S1. Linkage disequilibrium plot of 1504 biallelic sites across a ~399-kb genomic region at 1q42.2, based on datasets from JPT (Japanese in Tokyo) in the 1000 Genomes Project database. The position of SNPs examined is shown by the green arrow. The haplotype block was defined by the method of Gabriel *et al*. (2002) [36]. The genetic variants with the minor allele frequency of <0.01 were removed from the analysis.
