# Supplementary figures and images for: Association of EGLN1 genetic polymorphisms with SpO2 responses to acute hypobaric hypoxia in a Japanese cohort
Source: J Physiol Anthropol. 2018 Apr 6;37:9. doi: 10.1186/s40101-018-0169-7 (PMC5889538; doi:10.1186/s40101-018-0169-7)

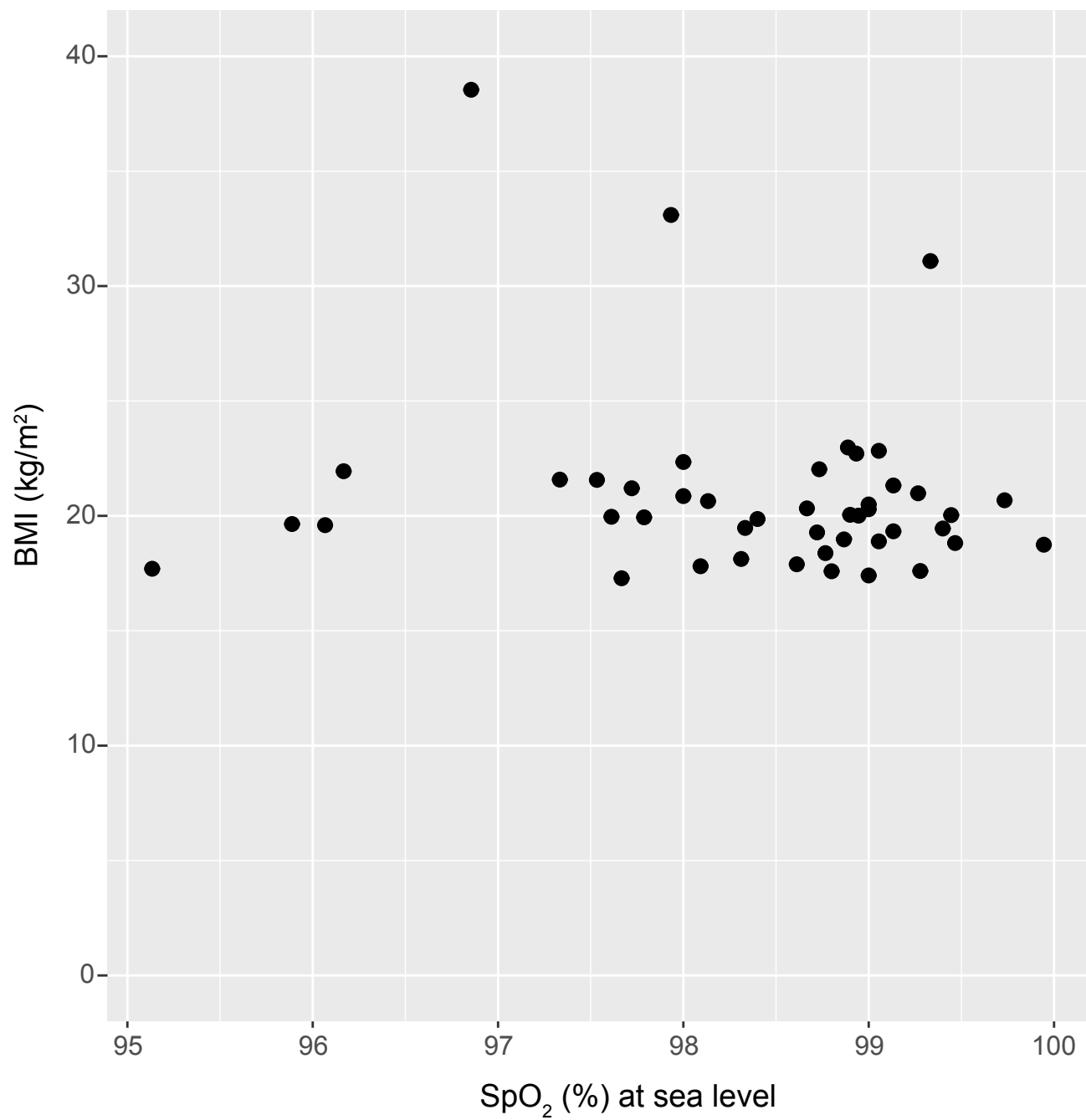

**Fig. S2.** Relationship between BMI and SpO<sub>2</sub> at sea level in 46 Japanese male students.

Supplement: Supplementary file 3 — Figure S2. Relationship between BMI and SpO2 at sea level in 46 Japanese male students. (PDF 101 kb) [file 40101_2018_169_MOESM3_ESM.pdf]
