# Supplementary material for: Association of EGLN1 genetic polymorphisms with SpO2 responses to acute hypobaric hypoxia in a Japanese cohort
Source: J Physiol Anthropol. 2018 Apr 6;37:9. doi: 10.1186/s40101-018-0169-7 (PMC5889538; doi:10.1186/s40101-018-0169-7)
Supplement: Supplementary file 4 — Table S2. Haplotype and diplotype frequencies of five SNPs around EGLN1 in 46 Japanese subjects. (PDF 71 kb) [file 40101_2018_169_MOESM4_ESM.pdf]

Table S2. Haplotype and diplotype frequencies of five SNPs around *EGLN1* in 46 Japanese subjects.

Haplotype	Observed number	Frequency	Diplotype	Observed number	Frequency
TCGGT	19	0.21	TCGGT/CTACC	8	0.17
CTAGC	14	0.15	TCGGT/CTAGC	5	0.11
CTACC	14	0.15	CTACC/CTAGT	4	0.09
TCGCT	13	0.14	TCGCT/TCGCT	4	0.09
CTACT	12	0.13	CTAGC/CTAGC	3	0.07
CTAGT	9	0.10	TCGCT/CTAGC	2	0.04
CTGGC	2	0.02	CTACT/CTAGT	2	0.04
TTGGC	2	0.02	CTACT/CTACT	2	0.04
CCAGC	2	0.02	TCGGT/CTACT	2	0.04
TCGCC	1	0.01	CTACT/CTGGC	1	0.02
TTACT	1	0.01	CTACT/CTAGC	1	0.02
TCACT	1	0.01	TCGCC/TCGCT	1	0.02
TTGGT	1	0.01	TTACT/CTACT	1	0.02
CTGCT	1	0.01	TTGGC/CCAGC	1	0.02
			TCACT/CTACT	1	0.02
			TCGGT/CCAGC	1	0.02
			CTAGT/CTAGT	1	0.02
			TCGCT/CTACC	1	0.02
			TTGGT/CTACC	1	0.02
			TCGGT/CTAGT	1	0.02
			TCGCT/CTGCT	1	0.02
			TTGGC/CTGGC	1	0.02
			TCGGT/TCGGT	1	0.02

Haplotype consists of five SNPs examined (from left, rs480902, rs479200, rs2808611, rs12097901, and rs2790859).
