# Supplementary material for: Association of EGLN1 genetic polymorphisms with SpO2 responses to acute hypobaric hypoxia in a Japanese cohort
Source: J Physiol Anthropol. 2018 Apr 6;37:9. doi: 10.1186/s40101-018-0169-7 (PMC5889538; doi:10.1186/s40101-018-0169-7)
Supplement: Supplementary file 5 — Figure S3. Relationship between SpO2 and minute ventilation at 60 min (equivalent to 4000 m) for rs12097901 (a) and rs2790859 (b). Red characters represent highlander alleles. Blue line and gray band represent a regression line and its 95% confidence interval, respectively. Circle colors indicate genotypes of each SNP. The mean slope of the regression line was − 0.018. Linear regression analysis showed no significant correlation (r2 = 0.001, P = 0.818). ANCOVA also showed no significant differences in the regression coefficient between genotypes (F(2, 40) = 1.21, P = 0.309 for rs12097901; F(2, 40) = 0.04, P = 0.961 for rs2790859) and in the adjusted mean values (F(2, 42) = 2.97, P = 0.062 for rs12097901; F(2, 42) = 0.11, P = 0.894 for rs2790859). (PDF 230 kb) [file 40101_2018_169_MOESM5_ESM.pdf]

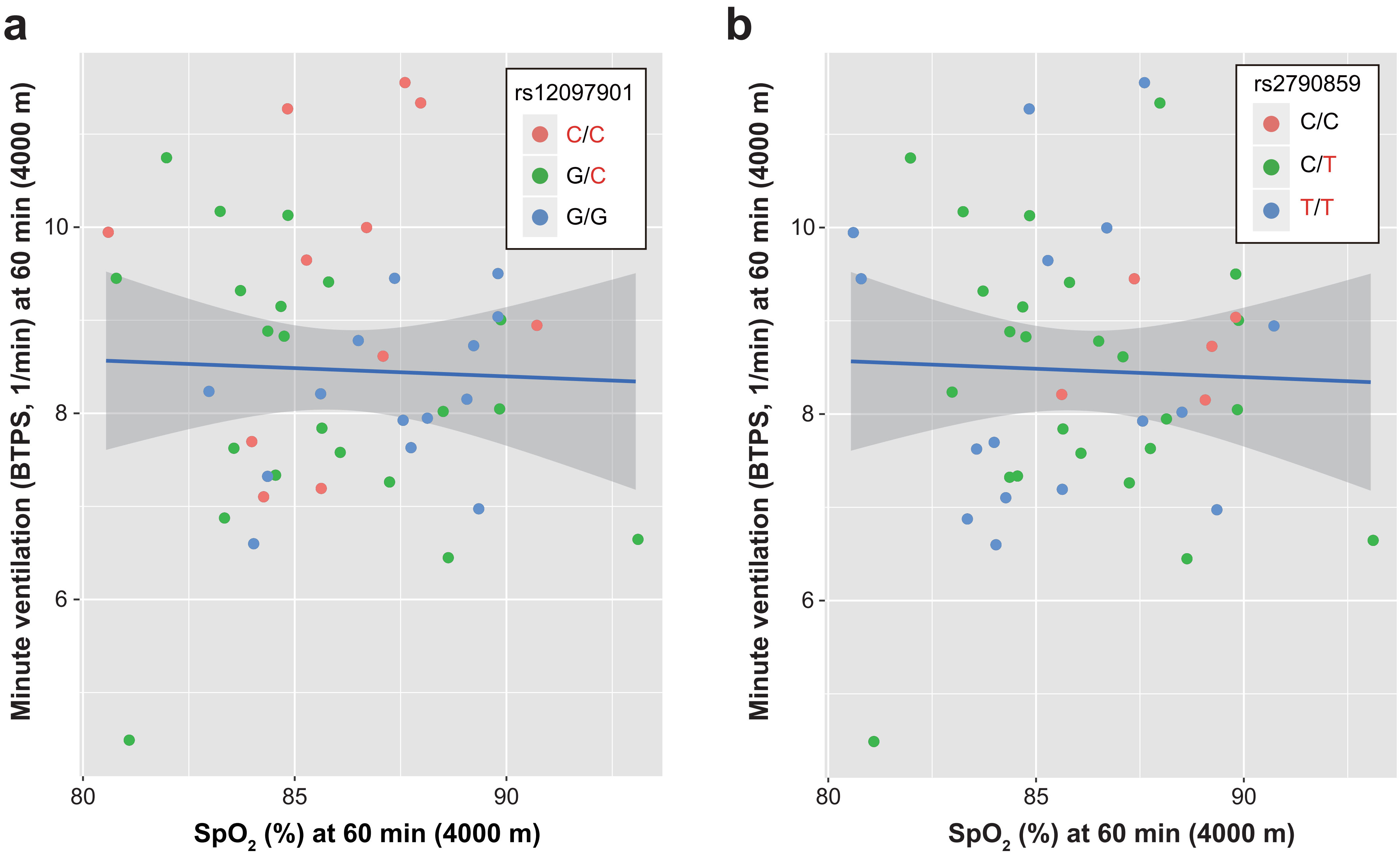

Fig. S3. Relationship between SpO_2 and minute ventilation at 60 min (equivalent to 4000 m) for rs12097901 **(a)** and rs2790859 **(b)**. Red characters represent highlander alleles. Blue line and gray band represent a regression line and its 95% confidence interval, respectively. Circle colors indicate genotypes of each SNP. The mean slope of the regression line was -0.018. Linear regression analysis showed no significant correlation ($r^2 = 0.001$, $P = 0.818$). ANCOVA also showed no significant differences in the regression coefficient between genotypes ($F_{(2, 40)} = 1.21$, $P = 0.309$ for rs12097901; $F_{(2, 40)} = 0.04$, $P = 0.961$ for rs2790859) and in the adjusted mean values ($F_{(2, 42)} = 2.97$, $P = 0.062$ for rs12097901; $F_{(2, 42)} = 0.11$, $P = 0.894$ for rs2790859).
