# Supplementary material for: Association of EGLN1 genetic polymorphisms with SpO2 responses to acute hypobaric hypoxia in a Japanese cohort
Source: J Physiol Anthropol. 2018 Apr 6;37:9. doi: 10.1186/s40101-018-0169-7 (PMC5889538; doi:10.1186/s40101-018-0169-7)
Supplement: Supplementary file 6 — Figure S4. Relationship between SpO2 and perfusion index at 60 min (equivalent to 4000 m) for rs12097901 (a) and rs2790859 (b). Red characters represent highlander alleles. Blue line and gray band represent a regression line and its 95% confidence interval, respectively. Circle colors indicate genotypes of each SNP. The mean slope of the regression line was 0.011. Linear regression analysis showed no significant correlation (r2 = 0.011, P = 0.481). ANCOVA also showed no significant differences in the regression coefficient between genotypes (F(2, 40) = 2.20, P = 0.124 for rs12097901; F(2, 40) = 0.69, P = 0.509 for rs2790859) and in the adjusted mean values (F(2, 42) = 1.29, P = 0.285 for rs12097901; F(2, 42) = 1.08, P = 0.348 for rs2790859). (PDF 227 kb) [file 40101_2018_169_MOESM6_ESM.pdf]

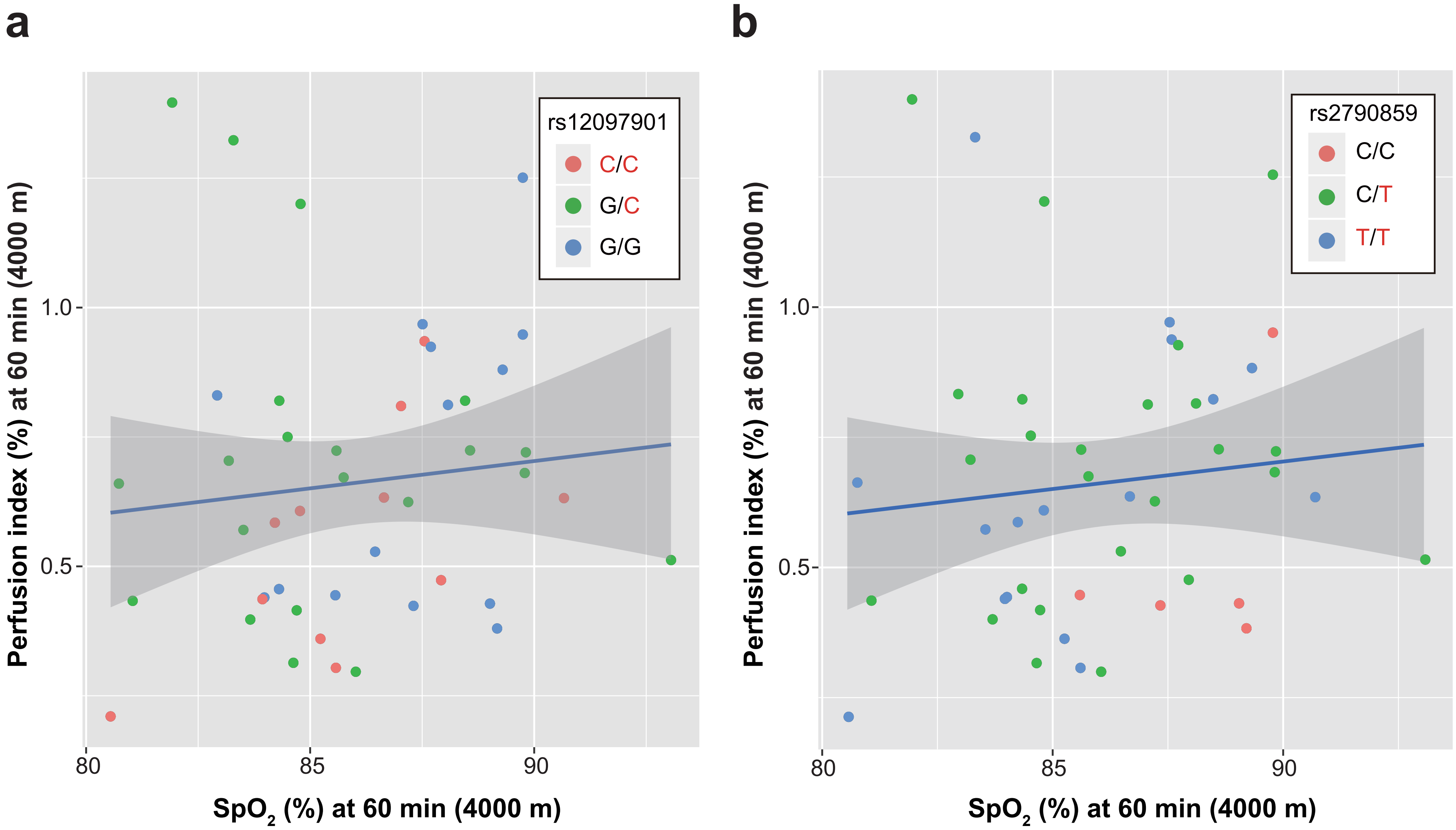

Fig. S4. Relationship between SpO_2 and perfusion index at 60 min (equivalent to 4000 m) for rs12097901 **(a)** and rs2790859 **(b)**. Red characters represent highlander alleles. Blue line and gray band represent a regression line and its 95% confidence interval, respectively. Circle colors indicate genotypes of each SNP. The mean slope of the regression line was 0.011. Linear regression analysis showed no significant correlation ($r^2 = 0.011$, $P = 0.481$). ANCOVA also showed no significant differences in the regression coefficient between genotypes ($F_{(2, 40)} = 2.20$, $P = 0.124$ for rs12097901; $F_{(2, 40)} = 0.69$, $P = 0.509$ for rs2790859) and in the adjusted mean values ($F_{(2, 42)} = 1.29$, $P = 0.285$ for rs12097901; $F_{(2, 42)} = 1.08$, $P = 0.348$ for rs2790859).
